# Stage-Dependent Tolerance of the German Cockroach, *Blattella germanica* for Dichlorvos and Propoxur

**DOI:** 10.1673/031.010.20101

**Published:** 2010-12-03

**Authors:** K. Qian, XQ. Wei, XP. Zeng, T. Liu, XW. Gao

**Affiliations:** ^1^ Department of Entomology, China Agricultural University, Beijing, 100193, China; ^2^ Beijing Center for Disease Prevention and Control, Beijing, 100013,China

**Keywords:** acetylcholinesterase, insecticide tolerance, stage-dependent susceptibility

## Abstract

Stage-dependent dichlorvos and propoxur tolerance in a field population of the German cockroach, *Blattella germanica* Linnaeus (Blatodea: Blattellidae), was investigated in the laboratory using a topical application bioassay. The results showed the 6 week-old nymphs were more tolerant to dichlorvos and propoxur than the other ages tested. LD_50_ values of dichlorvos and propoxur for the 6 week-old nymphs were 2.003 µµg per insect and 5.296 µµg per insect, respectively. Tolerance ratios of 18.55-fold and 4.98-fold for LD_50_ were obtained from 6-week-old nymphs compared to 4 week-old nymphs. The specific activity of acetylcholinesterase (AChE) from 1 week-old nymphs was the highest among all tested developmental stages of nymphs and adult males and females. The specific activity of AChE decreased significantly with increasing age. The sensitivity of AChE to dichlorvos was the highest with a *k*_i_ value of 3.12××10^4^ mol^-1^min^-1^ in the last nymphal stage of *B. germanica* (about 6 weeks-old). The AChE from 4 week-old nymphs was the most sensitive to propoxur, with the highest *k*_i_ value being 2.63××10^5^ mol^-1^min^-1^. These results indicated that the different developmental stages and sexes of *B. germanica* affected the inhibition of AChE by dichlorvos and propoxur.

## Introduction

The German cockroach, *Blattella germanica* Linnaeus (Blatodea: Blattellidae), is considered one of the most serious pests in the urban environment. In Beijing, large amounts of insecticides are applied annually in an effort to control this pest. However, *B. germanica* has developed resistance to most of the traditional insecticides (organochlorine, organophosphate, carbamate, pyrethroid) used for its control (Cornwell 1976; Cochran 1995).

Acetylcholinesterase is the target site for carbamate and organophosphate insecticides. The inhibition of AChE by carbamate or organophosphate insecticides occurs via a reversible complex formation followed by carbamylation or phosphorylation (Shi *et al.* 2002). AChE is a key enzyme in the transmission of nerve impulses, specifically in termination of cholinergic synaptic transmission in mammals and insects. The primary mechanism of acute toxicity of carbamate and organophosphate insecticides has been reported as its inhibition of AChE in the cholinergic synapse of the nervous system of the cockroach.

Many factors, such as developmental stage, sex, and reproductive and nutritional status, may influence the toxicity of insecticides to insects (Brattsten et al. 1973; Yu 1983). Usually adult male cockroaches were used as laboratory bioassays for evaluating insecticide resistance (Cornwell 1976; Koehler and Patterson 1988). However, some research indicated that mixed-sex, late-instar *B. germanica* nymphs (4–6 week-old instars) were significantly more tolerant to insecticides including pyrethroid, carbamate, and organophosphate insecticides than adult males in topical and residual insecticide bioassays (Koehler et al. 1993).

Organophosphate and carbamate insecticides have been widely used for *B. germanica* control in the last two decades in China (Zhang 1997; Lin 2000). *B. germanica* is the only major household insect that requires routine treatments with insecticide to prevent them from developing into excessively large pest populations. Age class distribution in natural populations of *B. germanica* demonstrated that nymphs comprised >80% of the populations (Reierson 1986; Ross and Mullins 1995). Difficulty in killing females and late-instar cockroach nymphs with insecticides has been reported from numerous laboratories (Koehler et al. 1993; Valles 1998). It is, therefore, necessary to study biochemical mechanisms of differential susceptibility of sexes and nymph age classes of *B. germanica* to insecticides. The inhibition of AChE is an important mechanism affecting the toxicity of organophosphate and carbamate insecticides to *B. germanica.*

This paper reports the investigation of the relationship between the stage- and genderdependent differential susceptibility and the inhibitory effects of dichlorvos and propoxur on acetylcholinesterase in *B. germanica.* The outcome of this study can be useful to increase the efficacy of the rational application of insecticides to control *B. germanica* in urban areas.

## Materials and Methods

### Insects

A field population of the German cockroach, *B. germanica,* was established from collections from urban dwellings in Beijing in 2003. The colony was maintained at 25 ±± 1°° C and 60% relative humidity under constant illumination. To acquire nymphs of a specific age, adult females with egg cases were reared in separate jars before experimentation. Nymphs were staged by age groups (Woodruff 1938; Koehler et al. 1993) where 2 week-old nymphs are primarily 3^rd^ instars, 4 week-old nymphs are 4^th^ and 5^th^ instars, 5 week-old nymphs are primarily 5^th^ instars, 6 week-old nymphs are primarily 6th instars. Weekly cohorts of nymphs and newly emerged (white) males and females were maintained in separate tubs and removed after anesthesia with carbon dioxide.

### Chemicals

Propoxur (99% pure) was purchased from Shandong Huayang Technology Co., Ltd., (www.huayang.com), and dichlorvos was purchased from Tianjin Qianjin Pesticide Factory (www.tjqianjin.com). Acetylthiocholine iodide (ATCh), 5, 5′?-dithiobis (2-nitrobenzoic acid) (DTNB), bovine serum albumin (BSA), and Triton X-100 were purchased from Fluka Chemical Co. (www.sigmaaldrich.com). All other chemicals used in the experiments were of analytical grade.

### Bioassay

Topical Assays: Cockroaches were anesthetized with CO2 and treated topically with dichlorvos or propoxur dissolved in 1µµl acetone. The dichlorvos or propoxur solutions were applied to the first abdominal sternite in five concentrations causing >0% and <100% mortality. Three replications containing 10 cockroaches per dose were conducted. Piperonyl butoxide (PBO; 100 µµg/per cockroach) was applied to the first abdominal sternite 2 hr prior to propoxur application. Mortality (no response to probing) was recorded 24 hr after treatment. Data were analyzed by probit analysis (POLO-PC, LeOra Software 1987).

### AChE preparation

All stages of *B. germanica* were frozen at -80°° C prior to biochemical assays. The whole bodies of 10 cockroaches were homogenized in ice-cold 0.1M phosphate buffer, pH 7.5, containing 1%(v/v) Triton X-100 (the amount of buffer was determined by the weight of each *B. germanica* specimen, 10 ml per gram), and the crude homogenate was centrifuged at 12,000 g for 30 min at 4°° C using an ASTEC Microtec 1524R centrifuge (ASTEC Co., Fukuoka Japan). The supernatant was used as an enzyme source for measuring AChE activity.

### AChE assay

AChE activity was measured using the method described by Ellman et al. (1961) with minor modifications by Gorun et al. (1978). Briefly, in a final volume of 0.2 ml, 100 µµl enzyme, and 100 µµl ATCh (5mM final concentration) was incubated at 30°° C for 15 min, the reaction was stopped with 3.6 ml of 0.125 mM DTNB- phosphate - ethanol reagent (12.4mg of DTNB dissolved in 125 ml 95% ethanol, 75 ml distilled water, and 50 ml 0.1M phosphate buffer, pH 7.5) as the thiol indicator. The color was read immediately at 412nm using a spectrophotometer (Lambda Bio 40, Perkin Elmer, www.perkinelmer.com). The control samples contained no enzyme during the incubation. After addition of the color reagent, appropriate amounts of enzyme solutions were added to the controls.

Protein content was determined by the method of Bradford (1976) using bovine serum albumin as a standard.

### Inhibition of AChE by inhibitors dependent on time

Inhibitors were pre-incubated with the enzyme, and then the mixture of inhibitors and enzymes were added to the tube containing the equivalent volume of substrate at 1 min intervals. The residual enzyme activity was measured and the percentage of inhibition was calculated compared with that of the control. Each assay was represented by three replicates. The final concentration of each inhibitor was 1 ×× 10^-5^ mol L^-1^.

### Determination of kinetic parameters

The apparent Michaelis-Menten constant (*K*_m_ ) and Maximal velocity (*V*_max_) for ATCh was determined by enzymatic activity measures at 11 substrate concentrations ranging from 0.2 mmol L^-1^ to 2.2 mmol L^-1^. Results comprise the mean values of three separate preparations with three determinations for 11 concentrations. The kinetic parameters, *K*_m_ and *V*_max_, were calculated with Enzifit software (Leatherbarrow 1987).

Three kinetic parameters, *k*_d_ (dissociation constant), *k*_2_ (phosphorylation rate constant or carbamylation rate constant), and *k*_i_ (bimolecular rate constant) were determined by methods described in Main (1964). Inhibition was measured in the absence of substrate by mixing inhibitor (dichlorvos and propoxur) in buffer with enzyme at 30°° C and the remaining activity was determined at 20 s intervals by adding 100 µµl portions to 100 µµl acetylthiocholine iodide (ATCh) according to the above method (2.5). A plot of the logarithm of residual activity (*v*_i_/*v*_o_) versus time was linear for a given inhibitor concentration. Bimolecular rate constants (*k*_i_) and phosphorylation (or carbamylationrate) rate constants (*k*_2_) were calculated by nonlinear regression using Enzifit software (Leatherbarrow 1987); the values of *k*_d_ were obtained from *k*_2_/*k*_i_. Assays were employed for at least five different concentrations for each inhibitor and three replicates were used per AChE group-inhibitor combination.

### Statistical analyses

All statistical tests were performed using the software InStat (GraphPad, www.graphpad.com). The data were statistically analyzed using one-way ANOVA and two-tail P value. The Tukey's test was used among the different stages of *B. germanica* in multiple comparisons with p <0.05.

## Results

### Insecticide bioassay

Toxicities of dichlorvos and propoxur to different stages of *B. germanica* were assayed (Table 1). For the two tested pesticides, LD_50_ increased significantly as nymphal age increased from 4 to 6 week-old nymphs and the male adults were more susceptible than the female adults. The 6 week-old nymphs showed the strongest tolerance to the two tested pesticides among all tested stages of *B. germanica.* LD_50_ of dichlorvos and propoxur in the 6 week-old nymphs reached 2.003 µµg per insect and 5.296 µµg per insect, respectively, which were 18.55- and 4.98-fold greater than LD_50_S in the 4 week-old nymphs.

The toxicity of dichlorvos and propoxur to different stages of *B. germanica* was synergized by PBO in different levels (Table 1). PBO reduced the tolerance level in different stages from 1.57- to 2.66-fold to dichlorvos and from 1.13- to 2.71-fold to propoxur, respectively. The lowest synergist of PBO for the two pesticides was found in 4 week-old nymphs (with synergistic ratios of 1.57-fold for dichlorvos and 1.13-fold for propoxur, respectively).

### Developmental changes of AChE activity

The activity of AChE was highest in newly hatched nymphs and decreased with age, except for 6 week-old nymphs in which the AChE activity was lower than male adults. The specific activity of AChE in newly emerged females was significantly lower than that of newly emerged males (Table 2).

### Inhibitory effect of dichlorvos and propoxur on AChE dependent on time

**Figure 1.**
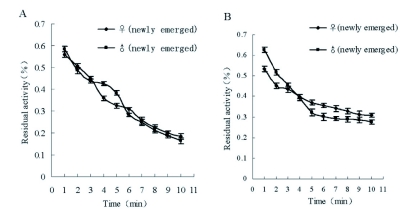
Inhibition of AChE progress with time in adult *Blattella germanica* (A: dichlorvos; B: propoxur). High quality figures are available online.

**Figure 2.**
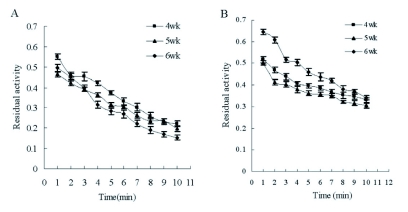
Inhibition of AChE progress with time in late-instar nymph of Blattella germanica (A: dichlorvos; B: propoxur). High quality figures are available online.

The similar inhibitory outline of AChE activity by dichlorvos or propoxur dependent on time was found between males and females (Figure 1). But the inhibitory time-course curve of propoxur showed that the residual AChE activity of adult males decreased more quickly than that of adult females. Figure 2 shows that there was no significant difference among the inhibitory effects of dichlorvos on AChE in last nymph stages (4–6 weeks-old); however the residual activity of AChE from 6 week-old nymphs decreased more quickly than that of the other nymph stages in the same interval after the treatment with propoxur.

**Table 1.**
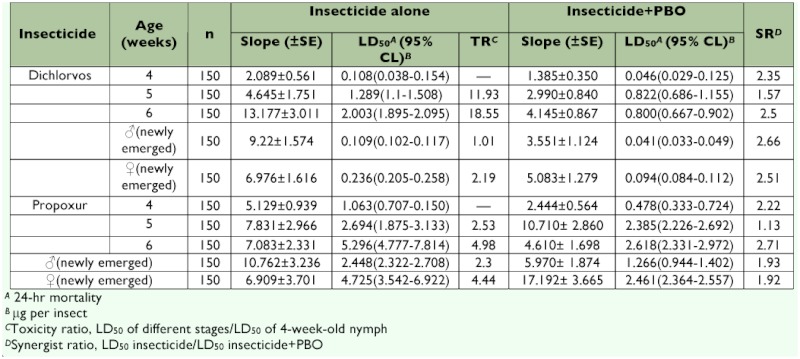
Toxicity of two insecticides with and without synergist to nymphal age and adults of *Blattella germanica.*

### Kinetics of AChE

The affinity of AChE for substrate, ATCh, of 6 week-old nymphs was the lowest and *K*_m_ values were significantly higher than those of other developmental stages (Table 3).

The sensitivity of AChE to dichlorvos in 6 week-old nymphs was the highest in all tested developmental stages with a *k*_i_ value of 3.12××10^4^ mol^-1^min^-1^, which was 2–3 times that of other stages of (Table 4). There was no significant difference in sensitivity of AChE to dichlorvos based on comparison of *k*_i_ value among the other tested ages. The highest affinity of AChE to dichlorvos was responsible for its highest sensitivity to dichlorvos, with a *k*_d_ value of 0.08; this was significantly less in 6 week-old nymphs than in other developmental stages, and the phosphorylation rate constant (*k*_2_) of AChE was also less in 6 week-old nymphs than in other developmental stages.

The sensitivity of AChE to propoxur in 4 week old nymphs was the highest in all tested developmental stages with a *k*_i_ value of 2.635××10^5^ mol^-1^ min-1 which was 2–3 times that of other stages (Table 5). A significant difference in *k*_i_ value was observed between the two sexes. The highest affinity of AChE to propoxur was responsible for its highest sensitivity to propoxur, with a *k*_d_ value of 0.004; this was significantly less in 4 week-old nymphs than in other developmental stages, and carbamylation rate constant (*k*_2_) of AChE was also less in 4 week-old nymphs than in other developmental stages.

**Table 2.**
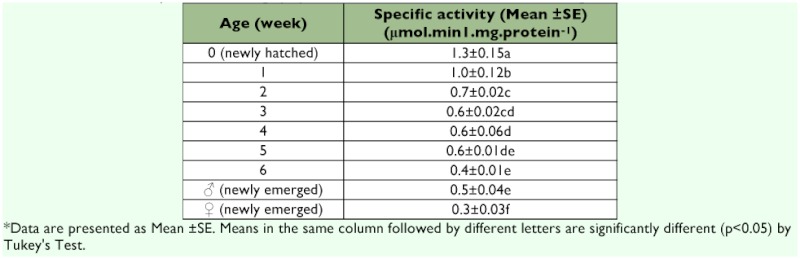
Relative activity of AChE among nymphs, adult females and adult males of Blattella germanica**

**Table 3.**
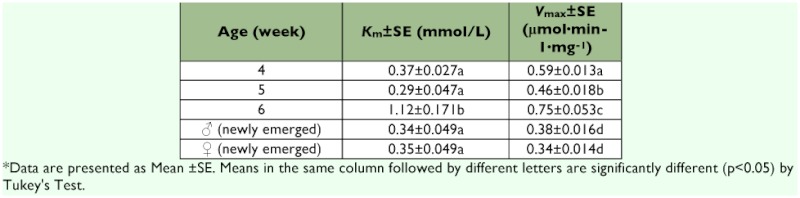
Michaelis-Menten constant (*Km*) and Maximal velocity (*Vmax*) of ATCh Hydrolyzed by AChE among nymphs, adult females and adult males of Blattella germanica**

### Discussion

Koehler *et al.* (1993) reported that late-stage nymphs of *B. germanica* were significantly more tolerant to bendiocarb, Cypermethrin, and chlorpyrifos than adult males. The present study obtained the same results for dichlorvos and propoxur. The 6 week-old nymphs were more difficult to kill than adult males and females in treatments with dichlorvos. The sensitivity of late-stage nymphs to propoxur was lower than that of adult males and almost same as adult females. This suggests that the pattern of tolerance to insecticides may be different among various stages of *B. germanica*. Bioassays for tolerance detecting in the laboratory may not necessarily reflect or predict control failure in the field due to differences in the nature of insecticide exposure in the field. However, they are useful for providing an indication of developmental tolerance changes in field populations of *B. germanica*.

**Table 4.**
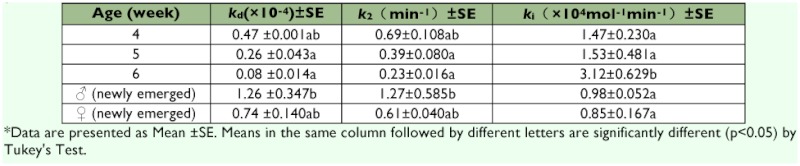
The kinetic constants of AChE inhibited by DDVP among nymphs, adult females and adult males of Blattella germanica^**^

**Table 5.**
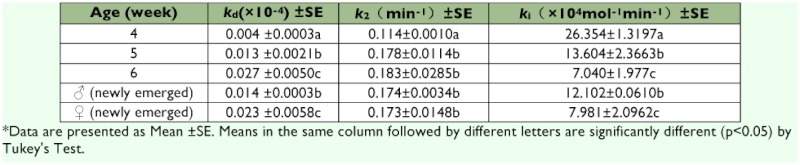
The kinetic constants of AChE inhibited by propoxur among nymphs, adult females and adult males of Blattella germanica**

Synergists, such as PBO, have been used widely against *B. germanica* to assess the contribution of metabolic insecticide resistance mechanisms (Valles et al. 1996). In the current studies, treatment of *B. germanica* with the cytochrome P450 monooxygenase inhibitor, PBO, increased dichlorvos and propoxur toxicity by 1.13- and 2.71-fold, respectively. Increased toxicity by the synergists suggested that cytochrome P450 monooxygenase was contributing to the detoxification of dichlorvos and propoxur.

Valles *et al.* (1994) showed the specific activity of AChE form adult males was 1.7 times higher than that of late-instar nymphs in the Village Green strain of *B. germanica.* But it was observed that the specific activity and Michaelis-Menten constants (*K*_m_) of AChE varied dependent on the ages of *B. germanica,* which indicates that characteristics of AChE can be different among the ages of *B. germanica.*

The consistent relationship between inhibition of AChE and the tolerance to dichlorvos was not observed in this study. However, the residual activity of AChE after the treatment with propoxur was higher in 6 week-old *B. germanica* than in 5 week-old and 4 week-old *B. germanica.* This suggests that inhibition of propoxur to AChE was weaker in 6 week-old *B. germanica* than in 5 week-old and 4 week-old *B. germanica.* The inhibition of AChE corresponded with the toxicity of propoxur to different ages of *B. germanica.*

Valles *et al.* (1996) reported that AChE sensitivity did not contribute to enhanced nymphal tolerance to propoxur; there were no significant differences in bimolecular rate constants for the inhibition of acetylcholinesterase by propoxur among adult males, final instar males, and female nymphs. In this study, the bimolecular rate constant (*k*_i_) for the inhibition of AChE by propoxur in 4 week-old nymphs was statistically higher than that in other tested stages. It was also consistent with the bioassay data of propoxur, indicating that higher sensitivity of AChE may be one of factors that are responsible for the more susceptibility of 4 week-old nymph to propoxur with a lower LD_50_ value.

The sensitivity of AChE from 6 week-old nymphs to dichlorvos was the highest in the tested nymph stages, but the bioassay data showed that the 4 week-old nymph was the most sensitive stage. It demonstrated that there was no direct relationship between the stage-and gender-dependent differential susceptibility and the inhibitory effects of dichlorvos on AChE in *B. germanica.*

Acetylcholinesterase is the target site for carbamate and organophosphate insecticides. The sensitivity of AChE to inhibitors varied with the different developmental stages of *B. germanica.* Therefore, selective application of insecticide for controlling *B. germanica* is very important based on developmental changes of AChE sensitivity to organophosphate and carbamate insecticides. However, it is impossible to explain the relationship between the inhibitory effects on AChE and the difference of insecticide susceptibility against developmental stages without the insecticide bioassay data. Results from the present study including biochemical and bioassay data, have demonstrated that stage-and gender-dependent differential susceptibility were consistent with the inhibitory effects of propoxur on acetylcholinesterase in *B. germanica,* but to dichlorvos there was no direct relationship observed.

In the present paper some preliminary results are reported on the relationship between the stage- and gender-dependent differential susceptibility and the inhibitory effects of dichlorvos and propoxur on acetylcholinesterase in *B. germanica.* Further work, including a better understanding of the molecular mechanisms that are operating, is necessary to improve control of *B. germanica* with organophosphate and carbamate insecticides.

## Abbreviations

AChE,: acetylcholinesterase;
**dichlorvos,**: the insecticide 2, 2-dichlorovinyl dimethyl phosphate;
**DTNB,**: Ellman'reagent 5, 5′?-dithiobis-(2-nitrobenzoic acid)
